# Kamishoyosan Alleviates Anxiety-like Behavior in a Premenstrual Syndrome Rat Model

**DOI:** 10.1155/2022/2801784

**Published:** 2022-10-14

**Authors:** Hikari Iba-Tanaka, Takuya Watanabe, Kyoka Harada, Kaori Kubota, Shutaro Katsurabayashi, Katsunori Iwasaki

**Affiliations:** ^1^Department of Neuropharmacology, Faculty of Pharmaceutical Sciences, Fukuoka University, 8-19-1 Nanakuma, Jonan-ku, Fukuoka 814-0180, Japan; ^2^A.I.G. Collaborative Research Institute for Aging and Brain Sciences, Fukuoka University, 8-19-1 Nanakuma, Jonan-ku, Fukuoka 814-0180, Japan

## Abstract

Kamishoyosan (KSS) is a traditional Japanese Kampo medicine that is prescribed for hormonal change-induced mood disorders including premenstrual syndrome (PMS). In clinical studies, KSS exhibited ameliorative effects on mood symptoms of PMS, such as anxiety and irritability. However, the mechanism underlying the beneficial effects of KSS is unclear. In the present study, we investigated the involvement of serotonergic machinery in the anxiolytic effects of KSS on hormonally-induced anxiety-like behavior in progesterone withdrawal (PWD) rats, which were used as a model of PMS. Female rats were injected with progesterone daily for 21 days. At 48 h after the final progesterone injection, anxiety-like behavior was evaluated using the elevated plus maze. KSS was administered orally to PWD rats 1 h prior to the test and significantly attenuated PWD-induced anxiety-like behavior. This ameliorative effect of KSS was reversed by WAY-100635, a serotonin (5-HT)_1A_ receptor antagonist. The effect of KSS on serotonergic transmission in the prefrontal cortex of PWD rats was also evaluated using an in vivo microdialysis procedure. KSS significantly increased the extracellular 5-HT level in the prefrontal cortex of PWD rats. In conclusion, our results suggest that KSS alleviates PWD-induced anxiety-like behavior at least partly by activating 5-HT_1A_ receptors and enhancing serotonergic transmission.

## 1. Introduction

Premenstrual syndrome (PMS) refers to various physical and emotional symptoms that occur during the luteal phase of each menstrual cycle [1]. Mood symptoms, such as anxiety, irritability, and mood lability, are the primary common symptoms and induce more severe impairment than physical symptoms [2]. Kamishoyosan (KSS), a traditional herbal medicine (Kampo medicine), is widely used to treat neuropsychiatric symptoms, such as mental anxiety and irritability, and physical symptoms associated with the changes in female hormones caused by menstruation and menopause [3–6]. Clinical studies have revealed that KSS has remarkable efficacy against PMS [3, 4, 7]. However, the precise mechanisms underlying the beneficial effects of KSS are unknown. Some mechanisms underlying the anxiolytic, antidepressive, and antiaggressive effects of KSS have been investigated in normal mice, social stress mice, and a postmenopausal mouse model [8–11]. However, no studies have investigated the mechanisms underlying the beneficial effects of KSS in rodent models of PMS.

Progesterone withdrawal (PWD) in rodents, which is produced by long-term exogenous progesterone injection followed by its abrupt cessation, has been established as a reliable animal model for mood disorders associated with PMS [12–14]. Serotonin (5-HT)_1A_ receptor activation was reported to attenuate depression-like behavior in the PWD model [13]. Although brain 5-HT dysfunction is believed to be associated with anxiety during PMS [15], it has not been determined whether serotonergic system activation can ameliorate anxiety-like behavior in the PWD model. We previously reported that PWD in rats sufficiently induced anxiety-like behavior in the elevated plus-maze (EPM) test, as well as PMS-like symptoms [16].

Thus, this study was designed to investigate the underlying mechanism by which KSS attenuates hormonal change-induced anxiety during PMS. Because the 5-HT_1A_ receptor is involved in anxiolytic effects, we examined the anxiolytic effect of KSS in PWD rats using WAY-100635, a selective 5-HT_1A_ receptor antagonist [17]. Furthermore, the effect of KSS on serotonergic transmission in the prefrontal cortex (PFC) was evaluated.

## 2. Materials and Methods

### 2.1. Animals

Female Wistar rats (6–7 weeks old) were purchased from CLEA Japan, Inc. (Tokyo, Japan). Rats were housed in groups of two to five per cage under a 12-h light/dark cycle (lights on between 07 : 00 and 19 : 00) at a temperature of 23 ± 2°C with a relative humidity of 60 ± 10% and allowed ad libitum access to food (CE-2, CLEA Japan, Inc.) and water. All animals were habituated to the maintenance condition for at least 1 week prior to starting the experiments. All animal care and use procedures were reviewed and approved by the Experimental Animal Care and Use Committee at Fukuoka University, Japan (^#^1911086).

### 2.2. Drugs

Dry powdered KSS extract (lot. no. 2160024010) was supplied by Tsumura & Co. (Tokyo, Japan). KSS comprises 10 dried extracts as shown in Table 1. Each plant component was authenticated by identifying the external morphology and marker compounds of plant specimens according to the methods of the Japanese Pharmacopoeia and the standards of Tsumura & Co. A mixture of 10 medicinal herbs was extracted with purified water at 95°C for 1 h. The extraction solution was separated from the insoluble waste and concentrated via the removal of water under reduced pressure. Spray drying was used to produce the dried extract powder. The yield of KSS extract was approximately 17.8%. The three-dimensional high-performance liquid chromatography (HPLC) profiles of KSS were previously presented [18].

KSS (100 and 1000 mg/kg, 10 ml/kg) was dissolved in distilled water. Buspirone hydrochloride (0.01 and 0.1 mg/kg, 1 ml/kg) and WAY-100635 maleate (0.3 mg/kg, 1 ml/kg), which were purchased from Sigma-Aldrich Co. (St. Louis, MO, USA), were dissolved in saline water. Progesterone and sesame oil were purchased from FUJIFILM Wako Pure Chemical Corporation (Osaka, Japan). The WAY-100635 concentration was selected based on the previous reports [19–21]. KSS was administered orally 60 min before the EPM test. Buspirone was administered intraperitoneally 30 min before the EPM test. WAY-100635 was intraperitoneally administered simultaneously with KSS 60 min before the EPM test. The same volume of vehicle was administered to control rats.

### 2.3. Rat PWD Model

**Figure 1:**
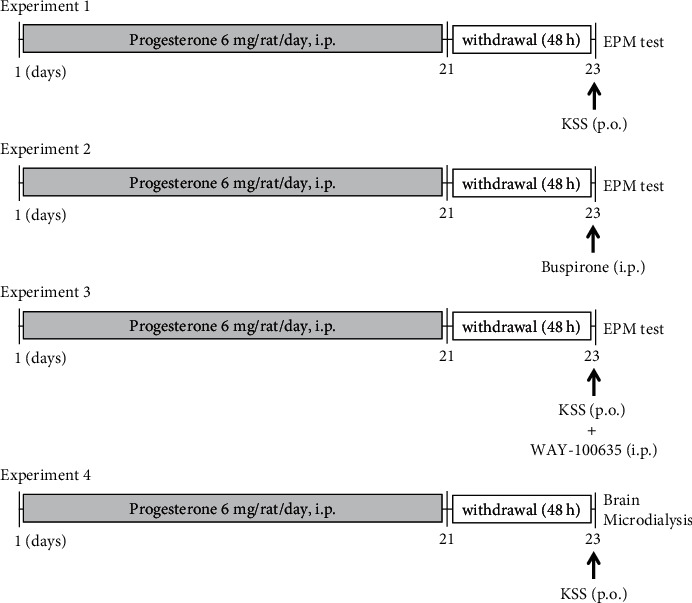
Experimental timeline for progesterone withdrawal, drug treatments, and behavioral tests. Experiment 1: examination of the effect of KSS on anxiety-like behavior induced by progesterone withdrawal. Female rats received daily progesterone injections (i.p.) for 21 days. The EPM test was performed 48 h after the final progesterone injection. KSS was administered p.o. 60 min before the EPM test. Experiment 2: examination of the effect of the 5-HT_1A_ receptor agonist buspirone on anxiety-like behavior induced by progesterone withdrawal. Buspirone was administered i.p. 30 min before the EPM test. Experiment 3: evaluation of the involvement of 5-HT_1A_ receptors in the effect of KSS on anxiety-like behavior. The 5-HT_1A_ receptor antagonist WAY-100635 was coadministered i.p. with KSS 60 min before the EPM test. Experiment 4: assessment of the effect of KSS on the extracellular 5-HT concentration. The implantation of a guide cannula for microdialysis was conducted 5–7 days before the microdialysis procedure. At 48 h after the final progesterone injection, dialysis probes were inserted into the guide cannula, and brain microdialysis was performed. After collecting dialysate samples for baseline measurements, KSS was administered p.o. EPM, elevated plus maze; KSS, Kamishoyosan; p.o., per os; i.p., intraperitoneally; 5-HT, serotonin.

PWD rats were generated according to a procedure described in our previous report [16]. All rats received daily intraperitoneal injections of progesterone (6 mg/rat/day suspended in 0.2 ml of sesame oil) or sesame oil (0.2 ml/rat/day) for 21 consecutive days. All injections were terminated 48 h before the behavioral test (Figure 1).

### 2.4. EPM Test

The EPM test was performed 48 h after the final progesterone injection (Figure 1) and was conducted according to a procedure described previously [16]. The apparatus consisted of two open arms (50 × 10 cm), two enclosed arms (50 × 10 × 45 cm), and a central platform (10 × 10 cm). The height of the apparatus was 50 cm above the floor. Each rat was placed on the central platform facing one of the enclosed arms and allowed to explore the maze freely for 10 min. The number of entries and the time spent in the enclosed arms were measured to determine the degree of motor function and anxiety, respectively [16].

### 2.5. *In Vivo* Microdialysis

#### 2.5.1. Surgery and Perfusion

Rats were anesthetized by intraperitoneal administration of a mixture of anesthetic agents (0.375 mg/kg medetomidine hydrochloride, Meiji Seika Pharma Co., Ltd., Tokyo, Japan; 2 mg/kg midazolam, Sandoz K. K., Tokyo, Japan; 2.5 mg/kg butorphanol, Meiji Seika Pharma Co., Ltd.), and a guide cannula (AG-4, Eicom Co., Kyoto, Japan) was stereotactically implanted in the PFC (in mm; anteroposterior +3.4, dorsoventral −4.0, and lateral −2.5 from bregma) using a method described by Hervas et al. [22] with modifications. The doses of anesthetic agents used in this study were determined according to the recommended anesthetics of the Research Institute National Center for Global Health and Medicine (Tokyo, Japan). Dummy probes were inserted into the guide cannula, and then rats were returned to their cages for 5–7 days to recover. Rats were housed individually after the operation.

Five to seven days after surgery and 48 h after the final progesterone injection, rats with sufficient postsurgical weight gain were selected for the studies. A dialysis probe with a membrane length of 2 mm (FX-I-4-02; Eicom Co.) was inserted into the guide cannula so that 2 mm of the dialysis membrane was exposed to the PFC tissue.

Experiments were performed in freely moving rats placed in a Plexiglas chamber (30 × 30 × 35 cm). After inserting the probes, perfusion was continuously maintained with Ringer's solution (NaCl, 147 mM; KCl, 4 mM; and CaCl_2_, 2.3 mM) at a flow rate of 1 *μ*l/min. At 4–4.5 h after starting perfusion, dialysate samples were collected at 30-min intervals in vials. After collecting four consecutive fractions to obtain basal values, a drug or vehicle was administered. Rats were only used for one experiment.

#### 2.5.2. Analytical Procedures for 5-HT

The 5-HT level in the dialysate sample was analyzed by an HPLC system with electrochemical detection (HPLC-ECD) (HTEC-500, Eicom Co.). A 20-*μ*l sample was injected into an HPLC-ECD system that included an EICOMPAK CAX column (2.0 mm i.d. ×200 mm, Eicom Co.) and was set at a potential of +450 mV against a Ag/AgCl reference electrode with a graphite carbon working electrode (WE-3G, Eicom Co.). The mobile phase consisted of 0.08 M sodium sulfate-0.1 M ammonium acetate buffer (pH 6.0) containing 30% (v/v) methanol and 50 mg/l Na_2_EDTA. Separations were conducted at 35°C with a flow rate of 0.25 ml/min. The standard 5-HT solution was injected every working day, and the amount of 5-HT in the samples was calculated on the basis of standard values using PowerChrom (version 2.2.4, Eicom Co.).

### 2.6. Statistical Analysis

All statistical tests were performed using GraphPad Prism 8 software (La Jolla, CA, USA). Results are expressed as the mean ± standard error of the mean (S.E.M.), and differences with a value of *P* < 0.05 were considered significant. One-way analysis of variance (ANOVA) followed by Dunnett's multiple comparison test or Tukey's multiple comparison test was used to assess the EPM test. For the microdialysis experiment, 5-HT levels are expressed as a percentage of the baseline level. Statistical analysis of the time course data was performed using two-way ANOVA (“time” as a within-subject factor and “treatment group” as a between-subject factor) followed by Tukey's multiple comparison test. The area under the curve data were analyzed using one-way ANOVA followed by Tukey's multiple comparison test.

## 3. Results

### 3.1. Effects of KSS on Anxiety-like Behavior Induced by PWD

**Figure 2:**
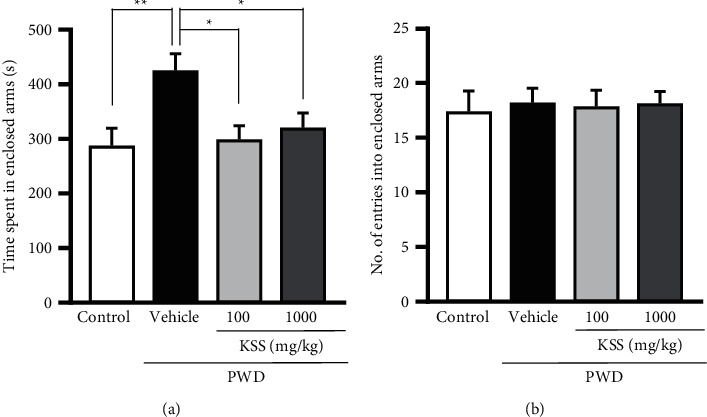
Effects of KSS on anxiety-like behavior in the EPM test during PWD. (a) The time spent in the enclosed arms and (b) the number of entries in the enclosed arms. KSS was orally administered 60 min before the EPM test. Values indicate the mean ± S.E.M. (^*∗∗*^*P* < 0.01, ^*∗*^*P* < 0.05). The control group, *n* = 10; the vehicle group, *n* = 9; the KSS 100 mg/kg group, *n* = 7; and the KSS 1000 mg/kg group, *n* = 8. KSS, Kamishoyosan; EPM, elevated plus maze; and PWD, progesterone withdrawal.

In experiment 1, we first investigated the effect of KSS on anxiety-like behavior in PWD rats using the EPM test. We previously reported that acute treatment with 100 mg/kg KSS prolonged the pentobarbital-induced sleep time in ovariectomized mice [18]. In this study, KSS was similarly administered at a dose of 100 mg/kg, and a 10-fold concentration of 1000 mg/kg was also examined to elucidate the dose-dependency of KSS. PWD rats spent more time in the enclosed arms of the EPM than control rats, which is indicative of anxiety-like behavior (*P* < 0.01, Figure 2(a)). KSS (100 or 1000 mg/kg) administration to PWD rats significantly reduced the time spent in the enclosed arms (*P* < 0.05, Figure 2(a)). Doses of 100 mg/kg and 1000 mg/kg of KSS showed similar efficacy. In addition, there was no significant difference in the number of entries in the enclosed arms among all groups (Figure 2(b)), demonstrating that motor function was not impaired. These results suggest that KSS has an anxiolytic-like effect without affecting the motor function in PWD rats.

### 3.2. Effects of a 5-HT_1A_ Receptor Agonist on PWD-Induced Anxiety-like Behavior

**Figure 3:**
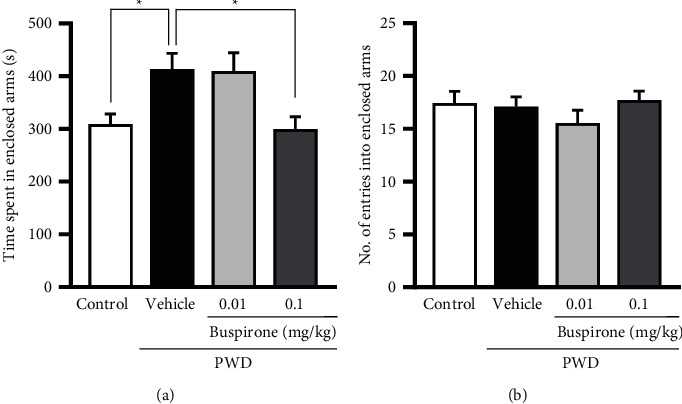
Effects of a 5-HT_1A_ receptor agonist (buspirone) on PWD-inducedanxiety-like behavior in the EPM test. (a) The time spent in the enclosed arms and (b) the number of entries in the enclosed arms. Buspirone was administered intraperitoneally 30 min before the EPM test. Values indicate the mean ± S.E.M. (^*∗*^*P* < 0.05). The control group, *n* = 11; the vehicle group, *n* = 12; the buspirone 0.01 mg/kg group, *n* = 11; and the buspirone 0.1 mg/kg group, *n* = 11. EPM, elevated plus maze; PWD, progesterone withdrawal; and 5-HT, serotonin.

To investigate whether 5-HT_1A_ receptor activation ameliorates PWD-induced anxiety-like behavior, we treated PWD rats with the 5-HT_1A_ receptor agonist buspirone in experiment 2. Administration of 0.1 mg/kg buspirone significantly shortened the PWD-induced increase in time spent in the enclosed arms of the EPM by PWD rats (*P* < 0.05, Figure 3(a)). There was no significant difference in the number of entries in the enclosed arms among all groups (Figure 3(b)). These results suggest that activating the 5-HT_1A_ receptor reduces anxiety-like behavior in PWD rats without affecting the motor function.

### 3.3. Involvement of the 5-HT_1A_ Receptor in the Ameliorative Effect of KSS on PWD-Induced Anxiety-like Behavior

**Figure 4:**
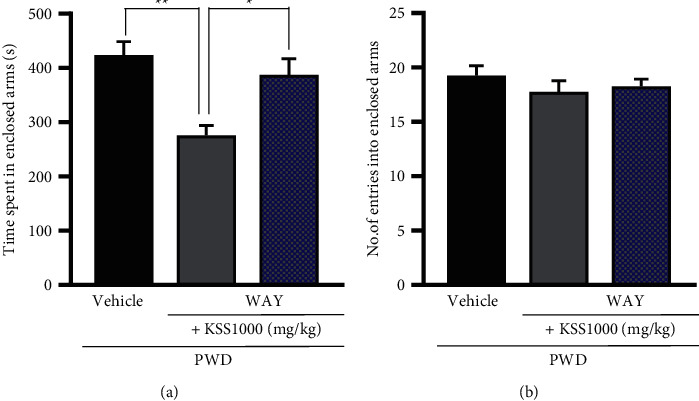
Effects of a 5-HT_1A_ receptor inhibitor, WAY-100635 (WAY), on the anxiolytic-like effect of KSS in the EPM test of PWD rats. (a) The time spent in the enclosed arms and (b) the number of entries in the enclosed arms. WAY was intraperitoneally injected when KSS (1000 mg/kg) was orally administered 60 min before the EPM test. Values indicate the mean ± S.E.M. (^*∗*^*P* < 0.05, ^*∗∗*^*P* < 0.01). The vehicle group, *n* = 8; the KSS1000 mg/kg group, *n* = 8; and the WAY + KSS 1000 mg/kg group, *n* = 8. KSS, Kamishoyosan; EPM, elevated plus maze; PWD, progesterone withdrawal; and 5-HT, serotonin.

Because 5-HT_1A_ receptor activation ameliorated anxiety-like behavior induced by PWD, in experiment 3, we next investigated whether agonistic action on the 5-HT_1A_ receptor was involved in the ameliorative effect of KSS on anxiety-like behavior during PWD. Although 100 mg/kg and 1000 mg/kg KSS showed similar effects on anxiety-like behavior (Figure 2(a)), the higher concentration of KSS (1000 mg/kg) was employed in this experiment to assess the serotonergic machinery. The selective 5-HT_1A_ receptor antagonist WAY-100635 was administered simultaneously with KSS. WAY-100635 (0.3 mg/kg) significantly reversed the KSS-induced reduction of the time spent in the enclosed arms (1000 mg/kg) by PWD rats (*P* < 0.05, Figure 4(a)). There was no significant difference in the number of entries in the enclosed arms among all the groups (Figure 4(b)). These results demonstrate that an agonistic action on 5-HT_1A_ receptors contributes to the anxiolytic effect of KSS during PWD.

### 3.4. Effects of KSS on the Extracellular 5-HT Concentration in the PFC of PWD Rats

**Figure 5:**
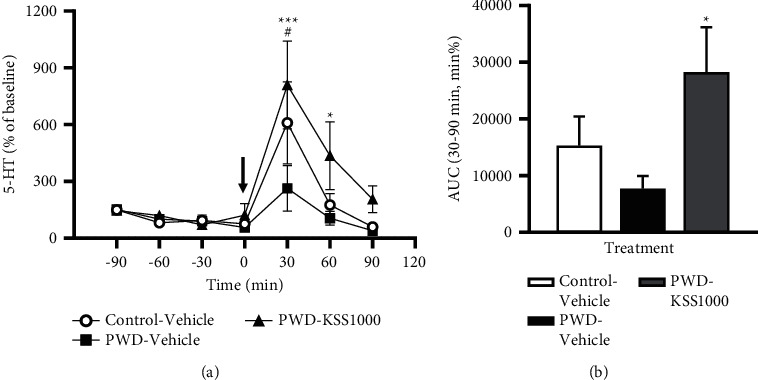
Effects of KSS administration on extracellular 5-HT levels in the frontal cortex of PWD rats. (a) Time course of extracellular 5-HT levels expressed as percentages of basal levels. The arrow indicates oral administration of 1000 mg/kg KSS (PWD-KSS1000) or distilled water (control-vehicle and PWD-vehicle) at time zero. Each point represents the mean ± S.E.M. (b) The area under the curve values calculated from 30 to 90 min. Each column indicates the mean ± S.E.M (min%). The control-vehicle group, *n* = 8; the PWD-vehicle group, *n* = 9; the PWD-KSS1000 group, *n* = 9. ^#^*P* < 0.05 and control-vehicle group vs. PWD-vehicle group; ^*∗*^*P* < 0.05, ^*∗∗∗*^*P* < 0.001, and PWD-vehicle group vs. PWD-KSS1000 group. KSS, Kamishoyosan; PWD, progesterone withdrawal; and 5-HT, serotonin.

To investigate the mechanism underlying the effect of KSS, we elucidated changes over time in extracellular 5-HT levels in the PFC of PWD rats in experiment 4. We found that 1000 mg/kg KSS transiently increased the extracellular 5-HT levels in PWD rats (Figure 5(a)). The extracellular 5-HT levels in PWD rats were significantly decreased 30 min after administration compared with those of control rats (*P* < 0.05, Figure 5(a)). Administration of KSS (1000 mg/kg) to PWD rats inhibited this reduction of 5-HT levels (*P* < 0.001Figure 5(a)). Overall two-way ANOVA showed a significant effect of treatment (*P* < 0.01) and an effect of time (*P* < 0.001, Figure 5(a)). As a result, the area under the curve of 5-HT levels calculated from 30 to 90 min after administration was also significantly increased in KSS-treated rats (*P* < 0.05, Figure 5(b)). These results suggest that KSS significantly increased the extracellular 5-HT level in the PFC of PWD rats, and this may also contribute to an anxiolytic effect of KSS during PWD.

## 4. Discussion

KSS is prescribed for PMS and has been reported to ameliorate mood symptoms [3, 4, 7]. However, the mechanism underlying the beneficial effects of KSS on PMS-related mood symptoms is unclear. The present study demonstrated that KSS attenuated PWD-induced anxiety-like behavior and increased extracellular 5-HT levels in the PFC of PWD rats. Furthermore, a 5-HT_1A_ antagonist attenuated the anxiolytic effect of KSS. Therefore, the anxiolytic effect of KSS may be mediated, at least in parts, by facilitating 5-HT release. The present study provides the first evidence that KSS ameliorates PWD-induced anxiety-like behavior by enhancement of serotonergic neurotransmission. In Japan, mood symptoms related to female hormone variation, such as that induced by the menstrual cycle and menopause, are called chi-no-michi-sho [23, 24]. The present study suggested that KSS attenuated hormonally-induced anxiety. Thus, KSS may be effective for chi-no-michi-sho.

Guo et al. demonstrated that KSS attenuates social phobia-related anxiety in ovariectomized mice and that the anxiolytic effect occurs because of facilitation of *γ*-amino butyric acid (GABA)_A_ receptor function [8]. Moreover, Egashira et al. reported that KSS potentiates pentobarbital-induced sleep in socially isolated, ovariectomized mice and that the effect involves benzodiazepine receptor function [18]. Thus, enhancement of the GABA-benzodiazepine system is suggested to be involved in the psychiatric effect of KSS in ovariectomized mice. However, Shimizu suggested that the antidepressive effect of KSS in chronically stressed, ovariectomized mice occurs because of amelioration of the 5-HT_1A_ receptor expression [11]. Thus, the mechanisms underlying the ameliorative effect of KSS in ovariectomized mice, which is a postmenopausal model, have previously been examined. However, there are no reports that investigate the mechanisms underlying the beneficial effects of KSS in PMS model rodents. The present study is the first to demonstrate that the mechanism underlying the anxiolytic effect of KSS in PMS model rats involves enhancement of serotonergic neurotransmission.

Although KSS increased the extracellular 5-HT levels in the PFC, we could not unveil the molecular mechanism by which this effect occurred. Tryptophan hydroxylase is the rate-limiting enzyme for 5-HT synthesis [25]. Administration of KSS to mice induces the gene expression of tryptophan hydroxylase in the dorsal raphe nucleus, which mainly contains serotonergic neurons [10]. In addition, albiflorin, which is a principal active ingredient of Paeoniae Radix and a major component of KSS, has been reported to increase the extracellular concentration of 5-HT in the rat brain via inhibiting the 5-HT transporter [26]. Therefore, KSS may enhance 5-HT synthesis and/or inhibit 5-HT reuptake, resulting in an increase in extracellular 5-HT levels. Vortioxetine acts as a 5-HT transporter inhibitor in addition to its effect as a 5-HT_1A_ receptor agonist [13]. Acute administration of vortioxetine during PWD reduces depression-like behavior [13]. These findings suggest that negative moods during PWD are attenuated by 5-HT_1A_ receptor activation and an increase in 5-HT in the synaptic cleft. Therefore, the present study demonstrates that KSS could be a valuable therapy for mood disorders during PMS.

## 5. Conclusions

Our findings suggest that treatment of premenstrual anxiety model rats with KSS produced anxiolytic effects that were mediated by enhanced 5-HT release in the PFC. The present study is the first to examine the mechanisms underlying the beneficial effects of KSS on premenstrual anxiety using a rodent model of PMS. Thus, our results provide important knowledge of how KSS relieves mood disorders in females experiencing PMS.

## Figures and Tables

**Table 1:** Formula of Kamishoyosan (KSS).

Plant names	Botanical origin	Weight (g)
Bupleuri radix	*Bupleurum falcatum* linne (root)	3.0
Paeoniae radix	*Paeonia lactiflora* pallas (root)	3.0
Atractylodis lanceae		
Rhizoma	*Atractylodes lancea* de candolle (rhizome)	3.0
Angelicae radix	*Angelica acutiloba* kitagawa (root)	3.0
Poria	*Poria cocos* wolf	3.0
Gardeniae fructus	*Gardenia jasminoides* ellis (fruit)	2.0
Moutan cortex	*Paeonia suffruticosa* andrews (bark)	2.0
Glycyrrhizae radix	*Glycyrrhiza uralensis* fischer (root)	1.5
Zingiberis rhizoma	*Zingiber officinale* roscoe (rhizome)	1.0
Menthae herba	*Mentha arvensis* var. *piperascens*	1.0

## Data Availability

The original contributions presented in the study are included in the article. Further inquiries can be directed to the corresponding author.
